# THE GRAYING OF PRIMARY CARE: THE ROLE OF PSYCHOLOGY IN GERIATRIC PRIMARY CARE

**DOI:** 10.1093/geroni/igz038.1428

**Published:** 2019-11-08

**Authors:** Erin Kube, Grant Harris, Bret Hicken

**Affiliations:** 1 Hines VAMC, Hines, Illinois, United States; 2 St. Louis VAMC, St. Louis, Missouri, United States; 3 SLC VAMC, Salt Lake City, Utah, United States

## Abstract

As of 2012, more than half of Veterans receiving care within a VA medical facility were age 65 or older. They have complex co-occurring medical and mental health needs, cognitive impairments, functional deficits, and psychosocial complexity. In 2015, GeriPACT emerged as a specialized geriatric primary care clinic model to serve this vulnerable population. The presence of psychologists in geriatrics has significant implications for treatment of cognitive, behavioral, and psychosocial needs. This mixed methods project aimed to describe the current scope and functions of GeriPACT psychologists and differentiate their services from other PACT clinics. Twenty total GeriPACT psychologists participated. The results suggest that mental health services within GeriPACT are multifaceted and need-driven. Significant themes highlight role specific characteristics of psychologists, clinician backgrounds, team education, and referral processes to improve access to care. Recommendations for implementation, clinician training, and future policy planning will also be presented.

